# Acute Onset of Peripheral Facial Nerve Palsy in Children: An Overview

**DOI:** 10.3390/pediatric16040072

**Published:** 2024-10-01

**Authors:** Virginia Fancello, Andrea Ciorba, Daniele Monzani, Elisabetta Genovese, Francesco Bussu, Silvia Palma

**Affiliations:** 1 Otolaryngology Division, Azienda Ospedaliero Universitaria Sassari, 07100 Sassari, Italyfbussu@uniss.it (F.B.); 2 ENT and Audiology Unit, Department of Neurosciences and Rehabilitation, University Hospital of Ferrara, 44121 Ferrara, Italy; 3 Unit of Otorhinolaryngology, Head and Neck Department, University of Verona, 37039 Verona, Italy; 4 Otolaryngology and Audiology Unit, University of Modena and Reggio Emilia, 41100 Modena, Italy; elisabetta.genovese@unimore.it; 5 Audiology, Primary Care Department, Modena AUSL, 41100 Modena, Italy

**Keywords:** peripheral facial nerve palsy, children, Bell’s palsy, acute onset, emergency department

## Abstract

**Background** The facial nerve (FN) plays a pivotal role in human life; apart from its sensory and parasympathetic functions, it innervates the facial muscles, and it is therefore involved in non-verbal communication, allowing us to express emotions and reactions. Especially in the case of childhood onset, FN dysfunction can severely affect the quality of life. **Methods** The aim of this review is to analyze the most recent literature, focusing on the acute onset of peripheral FN palsy among pediatric patients, discussing the different etiologies, prognoses, and management strategies. A total of 882 papers were initially identified, but only 7 met the selection criteria. Therefore, data on 974 children in total were pooled and analyzed. **Results** According to the findings of this review, FN palsy is idiopathic in most cases, while an infective etiology was identified as the second most common. The main pathogen agents identified were Borrelia Burgdorferi, especially in endemic areas, and Herpesviridae. Respiratory tract infections and/or ear infections were also described. Head trauma or direct injury of the FN accounted for 2% of all cases. **Conclusions** The overall FN recovery rate is high, even though the etiology remains unknown for most patients. Therapeutic indications are still lacking, especially in the case of non-recovering FN palsy. In our opinion, large, prospective studies are necessary for improving our knowledge of this disorder and establishing evidence-based approaches.

## 1. Introduction

The facial nerve (FN) is essential for several human actions such as speaking, swallowing, blinking, and non-verbal and emotional communication. FN impairment can have a severe impact on a subject, as individuals suffering from this dysfunction may develop dysphagia (particularly during the swallowing oral phase), which is associated with oral incontinence, dysarthria, difficulty breathing through the nose, and blink dysfunction. Therefore, quality of life can also be affected, as the alteration of one’s facial expression can be a major cause of distress, especially in public situations and specific contexts (i.e., school), severely interfering with sociality. In fact, particularly in the case of childhood onset, facial nerve palsy (FNP) is reported to impact the perception of feelings and emotions, eventually promoting the development of a sense of inadequacy and inferiority, with major psychosocial consequences [1]. Because of these implications, a prompt diagnosis of FNP is important, along with an eventual identification of its etiology, as it can be caused by different conditions, such as infections and middle-ear diseases but also autoimmune issues and malignancies. Outcomes and treatments may vary depending on the underlying etiology. Acute FNP is not a rare disorder at the pediatric emergency department, and Bell’s palsy, the most frequent condition, is a diagnosis of exclusion. The incidence of FNP varies across the human lifespan, affecting around 15–40 people per 100,000 adults [2], while among pediatric patients, it ranges from 2.7 to 10 people per 100,000 individuals depending on the age group, being less frequent in children younger than 10 years of age [3].

The aim of this paper is to review the recent literature on the acute onset of peripheral FN palsy among pediatric patients, discussing the different etiologies, prognoses, and management strategies.

## 2. Materials and Methods

This systematic review was performed by searching for English-language studies available on the Medline and Embase databases from 2014 through to 2024. The last literature search was completed in May 2024. The MeSH term “facial paralysis” was used as a keyword for the search. Further filters applied were an age range from 0 to 18 years old and papers in English.

Inclusion criteria were as follows:

o Studies involving >50 patients;
o Studies focusing on acute-onset FNP;
o Studies in English;
o Studies defining the FNP etiology.

Exclusion criteria were as follows:

o Studies involving <50 patients (a criterion developed to help detect studies with an appropriate sample size) [4];
o Studies evaluating only specific etiologies;
o Insufficient data (i.e., missing outcomes);
o Studies with duplicated data.

**Figure 1:**
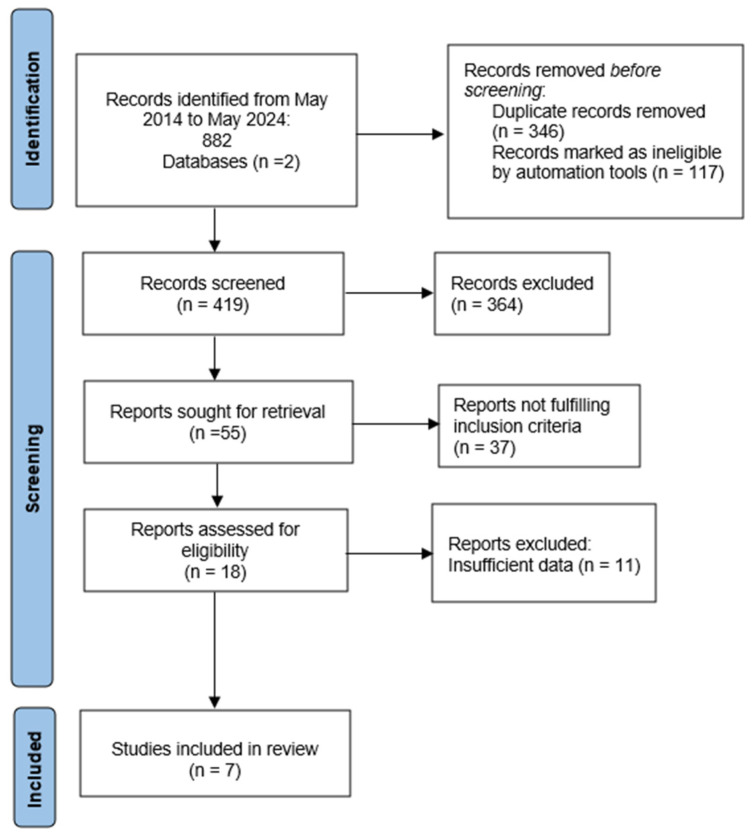
Study selection, as per PRISMA guidelines (http://www.prisma-statement.org/, accessed on 14 June 2024).

This review was completed according to the Preferred Reporting Items for Systematic Reviews and Meta-Analysis (PRISMA) guidelines. Figure 1 shows a corresponding flow diagram.

Two investigators assessed the data independently; the information, extracted from each selected paper, was then incorporated into an Excel database for further analysis.

## 3. Results

A total of seven papers met the above-mentioned criteria and were selected for analysis (see Table 1).

The studies were published online over a time span of 7 years, stretching from 2016 to 2023, whilst the included data were collected from 2006 to 2018. The information on 974 children was therefore analyzed: the M:F ratio was 0.8, and the mean age was 10.5, including children ranging from 1 to 18.

The clinical settings of the different studies were Pediatrics, Neurology, Infectious Diseases, and Ear, Nose, and Throat Departments, while the study design was retrospective in all the cases.

**Figure 2:**
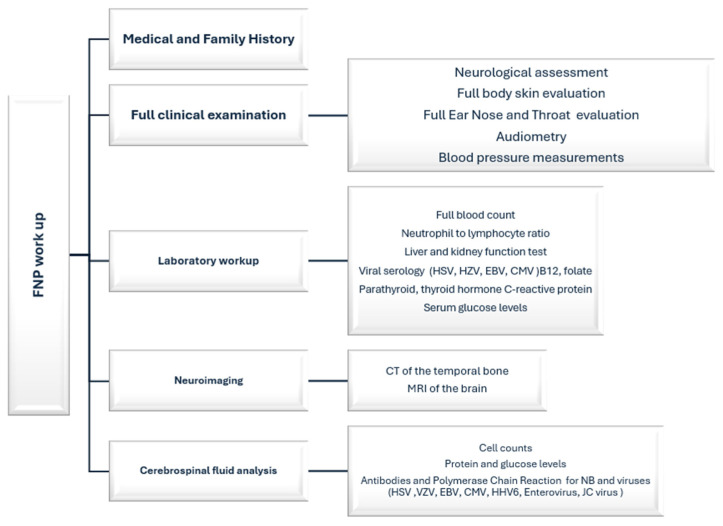
Diagnostic workups in the selected papers.

As defined in the inclusion criteria, the confirmed or supposed etiology was reported in each study for all cases. Within the selected papers, the diagnostic workup included accurate medical histories and clinical examinations. Further exams conducted were laboratory workups, neuroimaging, and, in case of signs of central nervous system involvement, a liquor analysis (see also Figure 2).

**Figure 3:**
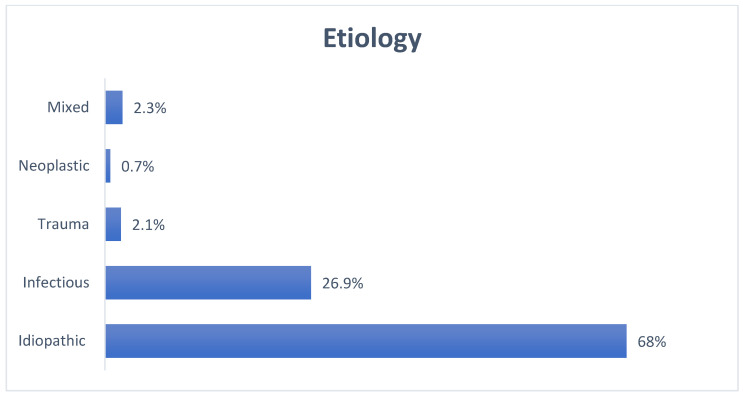
FNP etiologies—‘mixed’ includes iatrogenic damage, hypertension, drug side effects, and odontogenic causes.

**Figure 4:**
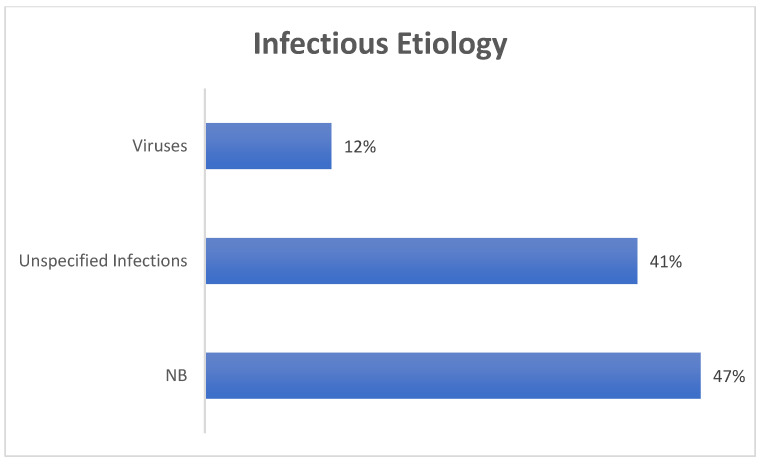
Infective etiology: viruses (HZV, HHV6, HSV, enterovirus, EBV, CMV); unspecified infection (including upper respiratory tract infection and ear infection not further specified); NB (neuroborreliosis).

FN palsy was defined as idiopathic in most of the cases (Bell’s palsy), while an infective etiology was identified as the second most common by all the authors (see also Figure 3 and Figure 4). Mixed etiologies included iatrogenic, syndromic, and cardiovascular causes. Neoplastic causes included solid brain tumors and leukemias.

Borrelia burgdorferi has been reported as the main agent causing FNP in endemic areas (in this review, in areas in the USA and Germany); however, upon considering regions where Lyme disease is uncommon, this percentage drops dramatically.

Among viruses, Herpesviridae (HZV, CMV, HSV, EBV) infections were the main etiologies, constituting the causative agent at a variable rate (up to 40% of the infective cases) within the included studies. Herpes zoster virus (HZV) was often responsible for FNP during primary infection or reactivation, as per Ramsay Hunt Syndrome.

Ear infections, with or without mastoiditis and concomitant respiratory tract infections (RTIs), were reported for 13% of children with FNP; surgical treatment was required only in complicated cases.

Head trauma or direct FN injuries were the other reported causes of FNP, accounting for 2% of all cases.

**Figure 5:**
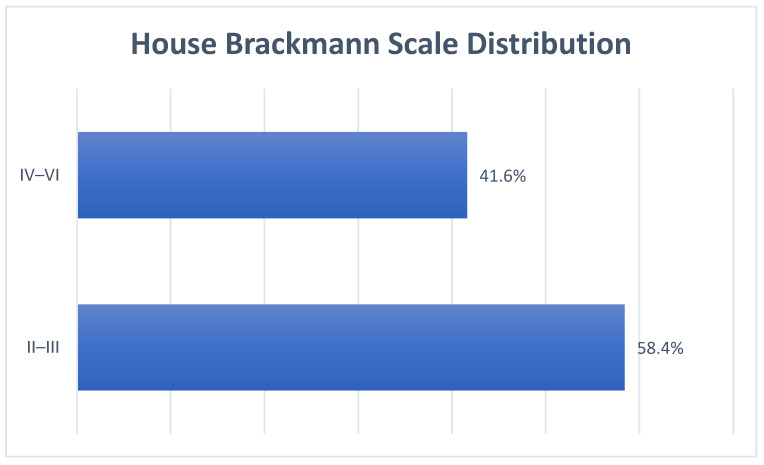
Overall House–Brackman scale distribution.

All the extracted studies except one [8] evaluated the degree of FN impairment using the House–Brackman scale (HBs). At the time of the first evaluation, most patients presented a mild to moderate degree of impairment (grade II–III for 58.4%—see Figure 5).

**Figure 6:**
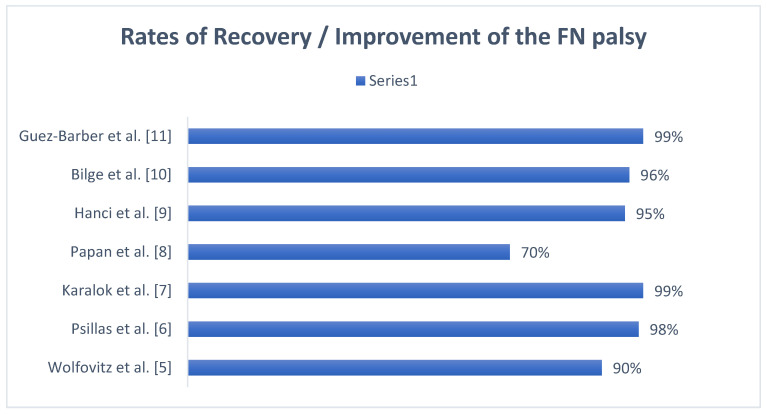
Rates of full recovery and/or improvement in each study. The rate reported by Papan et al. refers to the percentage of children who made a full recovery, whilst other authors reported the percentage of improvement in FN palsy [5,6,7,8,9,10,11].

The overall recovery rate was very encouraging, ranging from 70% to 99%, with a mean value of 95% (see also Figure 6).

Patients who were not recovering were affected by infectious diseases, benign and malignant tumors, or neurological disorders (including congenital conditions such as Melkersson–Rosenthal syndrome).

Overall, the long-term follow-ups revealed functional recovery within 6 months in most cases. However, the first months were reported to be crucial for the prognosis, especially for idiopathic palsy: in the series by Wolfovitz and Psillas [5,6], more than 90% of children fully recovered within the first two months since the onset.

Five of seven studies described a recurrence of FN impairment with a variable rate ranging from 4 to 11%.

Medical treatments of palsy included steroid therapy, associated with antiviral or antibiotic therapy in case of an underlining pathology, in particular acyclovir or valacyclovir in the case of HZV or other Herpesviridae and doxycycline for Lyme diseases. Patients with ear infections underwent antibiotic therapy, myringotomy, and, in some cases, mastoidectomy.

## 4. Discussion

This review fills the void of large prospective studies on FNP with an acute onset in children. In our opinion, if available in the future, this type of study could allow the formation of an enhanced approach to this pathology based on evidence and eventually tailored management (according to Patients’ features, etiology, etc.), especially for childhood cases.

According to the results of this review, the largest proportion of FNP cases is classified as idiopathic (Bell’s palsy), confirming previous findings [12]. To date, Bell’s palsy, in the case of adults, is still a diagnosis of exclusion. It is based on ruling out other possible causes of FNP, such as infections, trauma, neoplasms, and syndromic diseases through clinical, laboratory-based, and imaging evaluations [13]. The overall outcomes of this etiology were good in all the series, and steroids were the most reported treatment, with high rates of FN improvement, even if some patients developed aberrant nerve function after FNP, with subsequent synkinesis and hemifacial spasms. According to recent findings, children with Bell’s palsy can also improve without treatment; in particular, a multicenter study did not provide evidence that early treatment with prednisolone allows full FN recovery [14].

A small but not negligible share did not report substantial clinical recovery. The quality-of-life improvement among this subgroup of children is important; however, blink dysfunction is among the main concerns, and major complications of this condition are lagophthalmos, lower-eyelid ectropion, exposure keratopathy, corneal scarring, and subsequent visual impairment [15]. Lagophthalmos and ineffective Bell’s phenomenon are obvious risk factors for long-term sequelae [16]. Daily application of lubricants and eyelid taping at night are essential protective measures. Static procedures such as tarsorrhaphy and/or upper-eyelid gold or platinum weight implantation are advised in cases of persistent and incomplete recovery.

The second most frequent etiology of FNP in children is infectious disease. Among viruses, those in Herpesviridae, well known for their neurotropism, were the main causes according to the results of this review. In particular, among viruses within Herpesviridae, VZV is the most frequently involved agent, especially in Ramsay Hunt Syndrome. This syndrome is characterized by the reactivation of a latent VZV infection (even years after the primary infection) at the geniculate ganglion level. It manifests as herpetic vesicles in the external ear and/or oral mucosa, FN palsy, and a cochleovestibular deficit (eventually complicated by hearing loss and vertigo). The reported main therapy includes administration of oral steroids and Acyclovir of Valacyclovir (up to 30 mg/kg) and prednisone for 7 days (1–2 mg/kg/day at the beginning and then gradually tapered). In case of clinical features not consistent with a VZV reactivation, serological testing for herpes simplex virus, Epstein–Barr virus, and cytomegalovirus should be performed, even if their involvement is less frequent. A full neurological examination is necessary, as Herpesviridae infection may manifest with multiple cranial nerve impairments (especially cochleo-vestibular, trigeminal, glossopharyngeal, vagus nerve impairment) and life-threatening spreading as in the case of meningitis and encephalitis [17,18,19]. Nowadays, the consistent reduction (up to 80%) in varicella-related complications is related to the worldwide implementation of varicella vaccination [20,21].

Among viral etiologies, Sars-Cov-2 has also been proposed as a possible cause of FNP with an acute onset in children [22]; however, a clear cause–effect relationship has not been established yet [23].

In Borrelia Burgdorferi-endemic areas, the rate of FNP induced by a spirochete may reach up to 40% of all cases among children [24,25]. This diagnosis requires a combination of laboratory tests and clinical signs (such as migrant erythema and/or neurological involvement, including facial palsy). In areas where this disease is endemic, in case of high clinical suspicion, empirical therapy is recommended (as per the UK NICE guidelines) [26]. In addition, in endemic areas, this disease is most commonly present during the so-called “tick season”, during the warm months from April to October. The medical treatment includes the administration of antibiotics (i.e., doxycycline or amoxicillin); when the diagnosis and therapy are promptly made, the FNP outcome is usually good.

When acute FNP is related to the complication of an otitis, antibiotics and steroids are the treatment of choice. However, myringotomy and, in select cases, mastoidectomy may be necessary to improve the FNP course [27]. Myringotomy allows the draining of middle-ear secretions, eventually enabling one to take swabs for cultures and antibiograms.

FNP secondary to trauma can occur immediately or up to six days after the acute event, possibly due to the onset of a posttraumatic edema or the presence of bony spicules. A CT scan is essential for a correct assessment and, in particular, the identification of possible fracture lines. The most common traumatic injuries are reported to involve the geniculate ganglion region [28]. In this scenario, electroneurography (ENoG) and electromyography (EMG) are valuable diagnostic tools [29]. ENoG provides prognostic information on the condition of nerve fibers. The anterograde degeneration of axons, called Wallerian degeneration, will take 72 h to reach the extracranial portion of the nerve and further 21 h to its completion. Therefore, from the 3rd to 21st day, ENoG is the ideal prognostic tool [30]. On the other hand, muscle function can be evaluated through EMG, which is able to provide information on degeneration as well as reinnervation. In the case of FNP secondary to temporal bone trauma, it is very important to assess whether to proceed with facial decompression. Surgical management remains a controversial issue. However, many authors suggest that when ENoG and EMG indicate there is more than 90% degeneration of nerve fibers and denervation potentials, FN decompression should be performed [31].

Several neoplasms (leukemia, myelodysplastic syndromes, medulloblastoma, schwannoma, parotid gland tumors) have been reported to be the cause of FNP in a small proportion of children. Although tumors are rare events, accounting for less than 0.7% of cases in this review, a correct diagnosis is mandatory, particularly considering the critical relevance of early diagnosis for management and prognosis. Imaging, especially via MRI, is reported to be mandatory [32,33]; in case of schwannoma, a neurofibromatosis should be ruled out, and spinal cord MRI and genetic counseling are of paramount importance. A full blood count is essential to rule out leukemia or myelodysplastic syndromes. Peripheral blood analysis should be performed before the administration of steroids, which may alter the results, causing misdiagnoses.

Furthermore, there also are few rare conditions identified as causes of FNP in this review, such as Melkersson–Rosenthal syndrome. This is a congenital disease characterized by a delayed onset, usually in late childhood or adolescence [34]. The manifestations include recurrent or persistent orofacial edema (facial and lip edema), a fissured tongue, and relapsing, unilateral, or bilateral peripheral FN paralysis [35]. The diagnosis can be challenging, since most cases are paucisymptomatic without a simultaneous presence of the above clinical features. Nonetheless, it should be considered in the differential diagnosis, especially in cases of recurrent FNP, which were reported in three out of seven papers included in this review [7,9,10]. The outcome of FNP, especially when recurrent, is reported to be poor [36].

Finally, concerning the surgical treatment of FNP, it has been reported that in case of a direct nerve injury featuring an interruption of anatomical continuity, in children, early nerve grafting or reinnervation from a donor nerve, such as the masseterine branch of the mandibular nerve, is an optimal choice. The aim of this procedure is to restore the ability to produce a spontaneous smile, especially considering the well-known brain plasticity of children [37]. In cases without clear evidence of nerve anatomical interruption, if, after an appropriate follow-up of 6 to 12 months, investigations and neurophysiological studies exclude the possibility of spontaneous recovery, reanimation procedures should be considered. In the latter situations and in the event of congenital/central diseases with impairment of facial nucleus and/or long-standing denervation, muscle transfer techniques could be considered. Ideally, surgical rehabilitation aims at restoring either a symmetrical resting tone or appropriate spontaneous facial expressions.

Possible future treatments could include FN regeneration by means of various neurotrophic factors or stem-cell-based strategies; however, studies involving these possible forthcoming approaches are still in the preclinical stage [38]. Another final consideration is the FN grading system. To date, despite the diffusion of different grading systems such as Sunnybrook [39] or Sidney [40], the House–Brackman score remains the most shared and applied scale. In our opinion, a significant consensus on shared staging systems is favorable to improve research quality, as it can play a key role in an assessment, establishing prognosis, treatments, and follow-ups.

## 5. Conclusions

The assessment of peripheral FN palsy in children in case of an acute onset should always be accurate and detailed. The etiology of FNP still remains unknown in most cases (Bell’s palsy), even if the overall recovery rate of FN function is high. The management of the few children who do not exhibit spontaneous recovery can be particularly challenging, either due to some underlying etiologies or the clinical and cosmetic impact, especially when the palsy finally becomes permanent.

## Figures and Tables

**Table 1:** The table shows the selected papers. Main features of the selected studies; [R] = reference; R = retrospective; M = male; F = Female.

#	Author	[R]	Country	Study	Year	Mean Age	Sample	M	F
**1**	Wolfovitz et al.	[5]	Israel	R	2016	12 y	72	31	41
**2**	Psillas et al.	[6]	Greece	R	2018	9.9 y	124	49	75
**3**	Karalok et al.	[7]	Turkey	R	2018	10.77 y	144	65	79
**4**	Papan et al.	[8]	Germany	R	2019	10.9 y	158	80	78
**5**	Hanci F. et al.	[9]	Turkey	R	2019	9.7 y	113	55	58
**6**	Bilge et al.	[10]	Turkey	R	2022	9.6 y	57	25	32
**7**	Guez-Barber et al.	[11]	USA	R	2022	10.3 y	306	154	152

## Data Availability

Not applicable.
